# Separate and Unequal: The Cost of Coronavirus Disease 2019 on Childhood Health and Well-Being

**DOI:** 10.1089/heq.2020.0080

**Published:** 2021-02-25

**Authors:** Jared W. Magnani, Valerie Kinloch, Utibe R. Essien

**Affiliations:** ^1^ Department of Medicine, University of Pittsburgh School of Medicine, Pittsburgh, Pennsylvania, USA.; ^2^ Department of Medicine, Center for Research on Health Care, University of Pittsburgh School of Medicine, Pittsburgh, Pennsylvania, USA.; ^3^ School of Education, University of Pittsburgh, Pittsburgh, Pennsylvania, USA.

**Keywords:** childhood health, education, health outcomes

## Abstract

The coronavirus disease 2019 has and will have an untoward effect on children. In this perspective we summarize the short- and long-term impact of the pandemic on childhood social and physical health. School closure has resulted in an absence of educational opportunity, alongside deprivations of social structure, essential food, and adult guidance, as well as augmented deprivation for the neediest students. The loss of educational attainment will have long-term effects on social mobility, employment and income, and health outcomes. We advocate for transdisciplinary approaches and outline priorities to address the pandemic's impact on schools, literacy, and childhood welfare.

“There can be no keener revelation of a society's soul than the way in which it treats its children.”—Nelson Mandela

The coronavirus disease 2019 (COVID-19) has catalyzed the effects of social determinants on health disparities in the United States. There is a perception—suggested by both popular and scientific literature—that children have a relative immunity to the effects of COVID-19.^1,2^ Although evident that children differ substantively from adults with regard to transmission and symptoms,^3,4^ reassurances of childhood “immunity” from COVID-19 provide false comfort. Rather, many children facing the assault of this virus on their health will continue to experience its adverse effects for years to come. Even after the emergence of a vaccine and the resumption of a semblance of our prior normalcy, the public health and social welfare of today's vulnerable children is likely to resonate throughout their lifetimes. Figure 1 summarizes the toll of COVID-19 on children's well-being and suggests potential solutions.

**FIG. 1.**
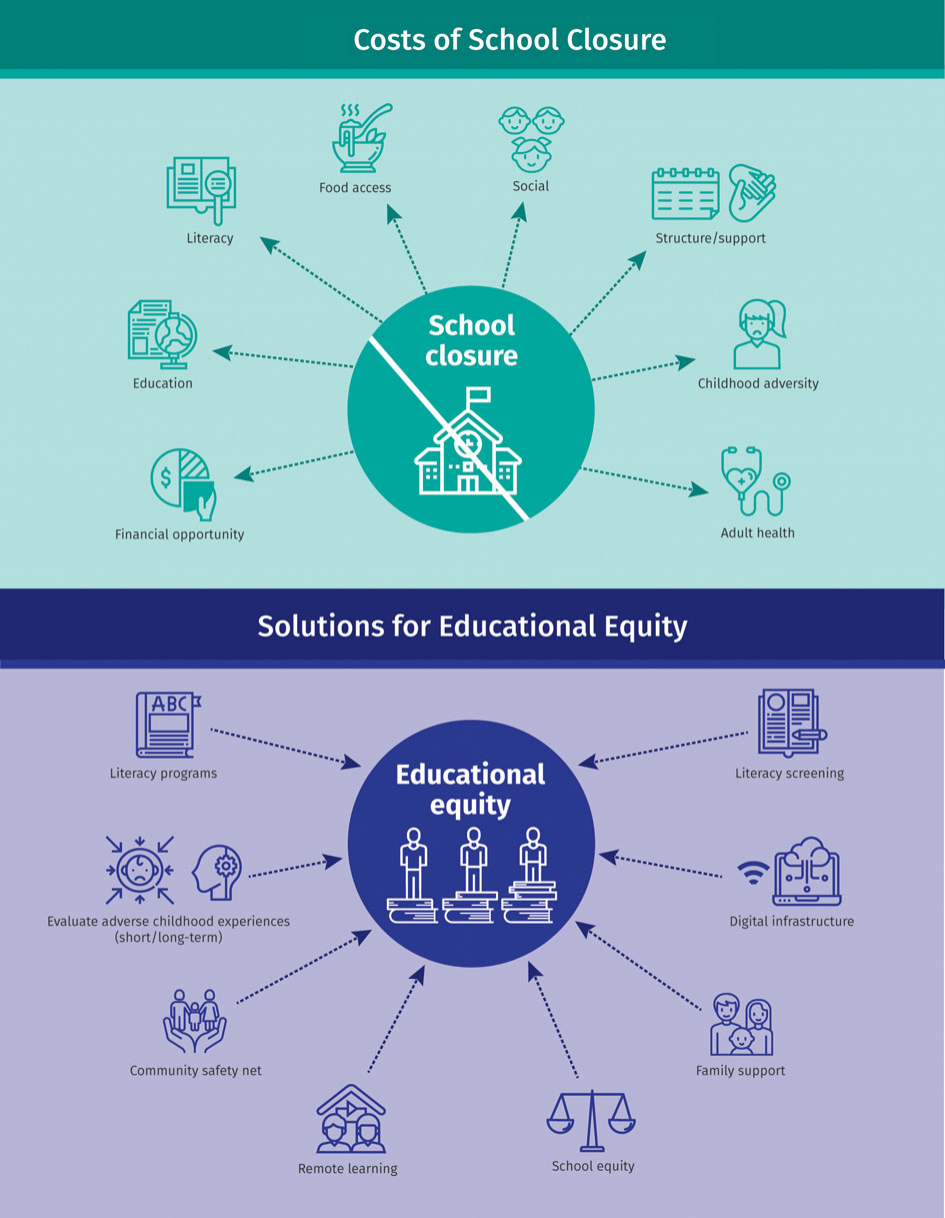
Summary of the multiple costs of school closure, extending from immediate- to long-term (*upper panel*), and the transdisciplinary solutions proposed to ameliorate and bolster educational equity (*lower panel*).

## School Closure and Educational Losses

The most evident cost to childhood well-being has been educational opportunity. Many school districts were not adequately prepared for the acute need to replace in-person instruction with digital learning. Those with limited resources faced the challenge of realistically retooling the classroom with a digital infrastructure at short notice. Many school districts as a result consigned themselves to scaled back expectations.^5^ For districts that lacked innovative leadership, budgets, and either capacity for digital classrooms or the expertise to implement such a transition, the decline in school-based educational engagement is no surprise. The duration of school closure remains uncertain, and problems present at the start of the pandemic remain unsolved.

The effects of school closures on students' learning are severe. For those struggling in primary and/or secondary school, a lost year yields an irrecoverable gap, and many can be expected to lose a full school-year worth of educational gain.^5^ Vulnerable students receiving limited support in schools with few resources will face increased likelihood of diminished academic success; educational deprivation begets educational failure. The loss of a full year of education has potential to impede academic advancement and produce long-term, possibly lifetime, socioeconomic repercussions. Such costs extend well beyond actual school re-entry.

The deeply fractured experience of justice, social opportunity, and health care access in our country is mirrored by schools. In the United States, public schools rely on property taxes, yielding immense discrepancies in resources, infrastructure, staffing, and class sizes, all relevant to academic achievement and advancement. Students residing in poorer districts—more likely to be minoritized or experience poverty—receive a categorically subpar education than their counterparts across town in wealthier neighborhoods. COVID-19 furthered the inherent inequities of our separate and unequal class-tiered educational system.

## Additional Effect of Poverty

School closure has augmented deprivations experienced by the neediest students. Children comprise 23% of the U.S. population, but 32% of those are living in poverty.^6^ For many students experiencing poverty, school can be a primary source of food, a refuge from homelessness, and a temporary destination of safety. The parents of children lacking economic security comprise the low-income “essential” workforce that keeps our society functional over the pandemic. Unmeasured but direct stressors on children's health stemming from COVID-related school closure are multiple: loss of the stability and social opportunity that school provides; deprivation of essential nutritional access; and absence of guidance and reassurance of a parent who cannot afford to shelter in place.

COVID-19 has further assaulted children in vulnerable families independent of school closure. The pandemic has wrought a cascade of job loss, erosion of families' financial security, and descent into poverty accompanied by food insecurity and loss of health insurance. The net yield is a social upheaval that is traumatizing for children. The biological and mental health effects of these myriad insults, unseen and unrecognized by our society, will impact the health of a generation.

## A Future in Doubt: Long-Term Health Impact

COVID-19 is a storm of collective insults to children's well-being that demarcate this present era as ripe with potential adversity. The long-term effects of COVID-19 on today's children are multiple. First, COVID-19 has threatened children's economic mobility. For vulnerable families, either minoritized or facing the complex challenges of being poor, educational attainment is the natural path toward social mobility. Individuals who attend college are more likely to find employment, purchase a home, and achieve financial security than those who do not. Educational achievement interrupts the generational poverty that marks the experience of many black and Latinx individuals and families in this country.

Second, a rich literature across multiple diseases demonstrates the association between educational attainment and health outcomes.^7^ One intermediate mechanism may be the increased health literacy associated with greater social and economic status.^8^ But a more evident yield from educational attainment is the resulting chain of opportunity: higher earning potential, residence in healthier neighborhoods, and improved access to health care and preventive services. Earning and household wealth are the mechanisms by which educational attainment improves health outcomes by facilitating health care access. A steep gradient exists in this country, the direct correlation between household wealth and life expectancy.^9^

Furthermore, education and wealth are generational. Children whose educational security and family's financial status are undermined by COVID-19 may experience the consequences over the next several decades. The events of the past few months can reinforce the well-trodden circular sequence of limited educational attainment, decreased earning potential and resulting financial insecurity, accompanied by limited access to health care and consequential decreased life expectancy.

Finally, the cascade of insults surrounding COVID-19 and school closure constitute an adverse childhood experience, recognized potential for its long-term repercussions on mental health, social well-being, and disease risk.^10^ Hence, the deprivations of COVID-19 on today's children are likely to extend and manifest into their adulthoods.

## COVID-19 and Childhood Costs: Old Challenges Demand New Solutions

Addressing the impact of COVID-19 on children needs far more than a familiar story of calling out racial, ethnic, and social disparities. There is no childhood immunity to the consequences from the assaults of this pandemic. In this moment of national reckoning with inequity and racism, we advocate for transdisciplinary approaches to measure and address the material, social, and educational deprivations experienced by vulnerable children. We highlight three areas to address the legacy inflicted by COVID-19 on children as summarized in Table 1.

**Table 1.** Priorities for Addressing the Effects of Coronavirus Disease on School-Age Children

Domain	Time frame	Priorities
School closure and childhood health	Short term	• Bolster digital infrastructure of under-resourced schools
		• Facilitate access to home-based technology and digital portals
		• Assess educational gaps, general literacy, and numeracy routinely as part of general health screening
Public health and public education	Intermediate	• Continue remedial education as part of health center mandate
		• Enhance school-based services to make up educational deficits
		• Channel resources for schools with limited resources to achieve academic benchmarks
		• Screen for adverse psychological and physical health effects of COVID-19 as part of childhood primary care
Literacy and research	Long term	• Promote literacy across early-, mid-, and late adulthood
		• Identify and examine long-term effects of adverse childhood experiences resulting from COVID-19

COVID-19, coronavirus disease 2019.

First, educational equity is paramount. We need immediate focused interventions to assist schools struggling with creating a digital environment. This requires developing and implementing best practices for remote learning and building safety nets to make sure that the pandemic leaves no child behind. Our society's attention to social media and mobile entertainment needs to be funneled toward a national agenda to promote equitable online learning during this pandemic. It has long been unconscionable that childhood education should suffer because of the absence of neighborhood wealth. Such an initiative will have long-lasting effects by assisting highly under-resourced schools to achieve modern digital infrastructure.

Second, we advocate for integrated services to address educational challenges. Limited literacy is an objective risk factor for adverse health outcomes. Children should be screened for literacy just as they are for exposure to firearms and smoking. In community settings, literacy screenings, interventions, and relevant support are critical to bridge educational gaps propelled by COVID-19.

Finally, the long-term effects of COVID-19 on children and childhood merit investigation. There are large questions about the immediate effects of social disruption, isolation, and food and financial insecurity. There are also longer term psychological, behavioral, and other health effects over the life course secondary to this adverse childhood experience. We advocate for an innovative transdisciplinary public health agenda to address the significant deprivations that have resulted from the pandemic.

## Conclusion

In summary, COVID-19 has had enormous impact on childhood well-being. Children, in no way immune to this virus, will face its horrendous adversity for their entire lifetime. Allied health professionals, along with policymakers and education leaders, must collaborate to achieve a future of equitable education and health.

## Abbreviation Used

COVID-19: coronavirus disease 2019
